# Potential bioactive coating system for high-performance absorbable magnesium bone implants

**DOI:** 10.1016/j.bioactmat.2021.10.034

**Published:** 2021-10-27

**Authors:** Murni Nazira Sarian, Nida Iqbal, Pedram Sotoudehbagha, Mehdi Razavi, Qamar Uddin Ahmed, Cortino Sukotjo, Hendra Hermawan

**Affiliations:** a Institute of Systems Biology (INBIOSIS), Universiti Kebangsaan Malaysia, 43600, Bandar Baru Bangi, Selangor, Malaysia; b Bio-Medical Engineering Centre, University of Engineering & Technology Lahore, New Campus, Pakistan; c BiionixTM (Bionic Materials, Implants & Interfaces) Cluster, Department of Internal Medicine, College of Medicine, University of Central Florida Health Sciences Campus at Lake Nona, 6900 Lake Nona Blvd, Orlando, FL 32827, USA; d Department of Pharmaceutical Chemistry, Kulliyyah of Pharmacy, International Islamic University Malaysia, 25200, Kuantan DM, Pahang, Malaysia; e Department of Restorative Dentistry, College of Dentistry, University of Illinois at Chicago, USA; f Department of Mining, Metallurgical and Materials Engineering, Laval University, Quebec City, G1V 0A6, Canada

**Keywords:** Absorbable metals, Bioactive agent, Bone fracture, Coating, Magnesium alloys

## Abstract

Magnesium alloys are considered the most suitable absorbable metals for bone fracture fixation implants. The main challenge in absorbable magnesium alloys is their high corrosion/degradation rate that needs to be controlled. Various coatings have been applied to magnesium alloys to slow down their corrosion rates to match their corrosion rate to the regeneration rate of the bone fracture. In this review, a bioactive coating is proposed to slow down the corrosion rate of magnesium alloys and accelerate the bone fracture healing process. The main aim of the bioactive coatings is to enhance the direct attachment of living tissues and thereby facilitate osteoconduction. Hydroxyapatite, collagen type I, recombinant human bone morphogenetic proteins 2, simvastatin, zoledronate, and strontium are six bioactive agents that show high potential for developing a bioactive coating system for high-performance absorbable magnesium bone implants. In addition to coating, the substrate itself can be made bioactive by alloying magnesium with calcium, zinc, copper, and manganese that were found to promote bone regeneration.

## Highlights

• Bioactive-coated magnesium implant could accelerate bone fracture healing time to match with magnesium degradation.
• Hydroxyapatite, collagen type I, recombinant human bone morphogenetic proteins 2, simvastatin, zoledronate, and strontium are high potential bioactive coating materials.
• The incorporation of Ca, Zn, Cu, Sr, and Mn in Mg base-metal could further enhance bone formation.

## Graphical abstract

**Figure:**
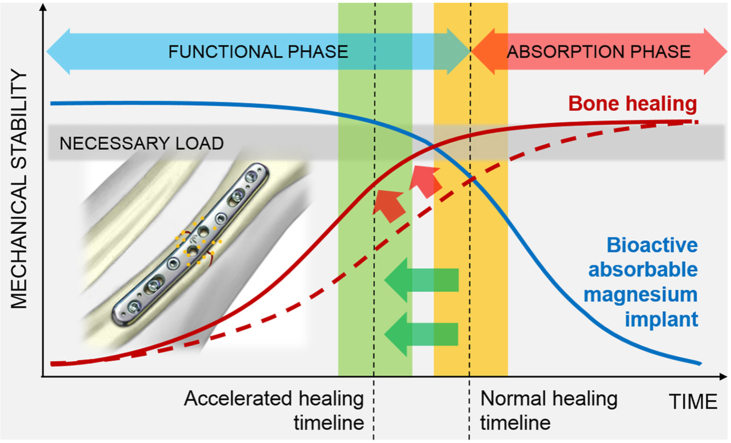

## 1 Introduction

**Fig. 1:**
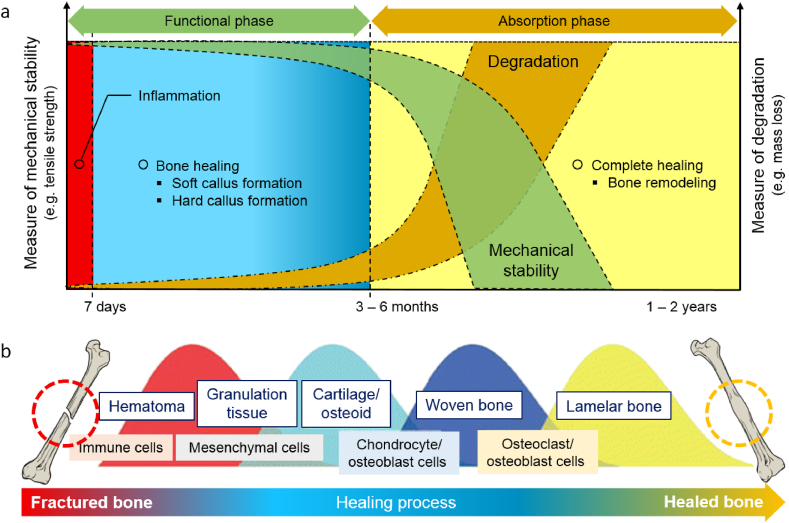
(a) Illustration of the ideal compromise between mechanical stability and degradation of absorbable metals for a bone screw, where the degradation rate stays low during the first 3–6 months while the mechanical stability stays high to support bone healing; (b) illustration of bone fracture healing process showing the four consecutive phases of healing, adapted from Ref. [4]. Within 1–7 days, an inflammatory response starts together with the formation of hematoma, resulting in the development of granulation tissue. Within 2–4 weeks, soft cartilage grows from the granulation tissue that further develops into a hard-bony callus that surrounds the fracture in 2–4 months. Within a few months to years, ossified callus regenerates to match the original bone morphology. *Colors indicate the healing process.

Biodegradable (absorbable) metals are designed to degrade in the human body (*in vivo*) via an electrochemical mechanism of metal dissolution (corrosion or degradation) and then metabolized or assimilated by cells and tissue [1,2]. These metals are intended to be used as medical implants that provide temporary mechanical support during the healing process of damaged tissue, such as a bone fracture. Therefore, an ideal absorbable metal implant must degrade properly without inducing any undesirable reaction in the host and should be absorbed once the healing process is completed [3]. As illustrated in Fig. 1, while degrading, the implant must maintain its mechanical integrity until the tissue regains its strength; therefore, its degradation rate must match the required bone healing period, i.e., 3–6 months for bone healing.

Among the studied absorbable metals, magnesium (Mg) and its alloys are considered the most promising candidates. They have been applied for making bone pins and screws, surgical clips, wires, sutures, and coronary stents [[5], [6], [7], [8]]. A notable challenge in using Mg and its alloys is their relatively high degradation rates [9]. Most research studies have been focused on controlling their composition, microstructure, and processing to arrive at new alloys with degradation rates that match the healing period [2,5]. Some studies developed coatings and surface treatments to regulate the degradation rate, i.e., by postponing the start of the degradation process [10,11]. On the other hand, some focused on adding bioactive agents to target clinical events associated with implantation, such as hydroxyapatite (HA) for enhancing bone regeneration and silver for improving antibacterial activity [12,13].

In the electrochemical corrosion reaction of Mg alloys, Mg cations form an anodic reaction that generates electrons (Eq. (1)). These electrons will be used in the anodic reaction of water reduction (Eqs. (2), (3))) followed by Mg hydroxide formation (Eq. (4)) [14]:

(1) $$Mg\to Mg^{2+}+2e^{-}$$

(2) $$2H_{2}O+2e^{-}\to H_{2}+2OH^{-}$$

(3) $$2H_{2}O+O_{2}+4e^{-}\to 4OH^{-}$$

(4) $$Mg^{2+}+2OH^{-}\to Mg{\left(OH\right)}_{2}$$

The Magnesium hydroxide is unstable in presence of chlorine ions and will be dissolved (Eq. (5)) [15]:

(5) $$Mg{\left(OH\right)}_{2}+2Cl^{-}\to MgCl_{2}+2OH^{-}$$

Also, the high chlorine concentration can cause pitting and localized corrosion in Mg alloys, leading to the early implant fracture and therefore should be controlled [16]. However, *in vivo* degradation is more complex due to protein, enzymes, cells, etc. Besides the decrease in the mechanical properties due to the high corrosion rate of Mg alloys, the hydrogen evolution and alkalization should be reduced. Hydrogen generation can form a gas pocket in the surrounding tissue that inhibits ideal implant/tissue interaction and the occurrence of tissue necrosis [17]. The latter causes an increase in pH (over 7.8), and alkalization poisoning would induce toxicity [18]. There are several studies that investigated the effect of alloy design on corrosion control, including the use of calcium (Ca) [[19], [20], [21]], zinc (Zn) [[22], [23], [24]], gadolinium Gd [[25], [26], [27]], manganese (Mn) [[28], [29], [30], [31]], strontium (Sr) [30,31], lithium (Li) [32,33], yttrium (Y) [[34], [35], [36], [37]], and zirconium (Zr) [37,38]. It should be noted that the amount of second phase formation and its distribution and microstructural evolution can significantly influence the corrosion rate of Mg alloys [39]. Furthermore, the amount of alloying elements should be controlled to inhibit toxicity. On the other hand, some studies show the corrosion rate of Mg alloys can be controlled by mechanical deformation such as rolling and equal channel angular pressing, due to their influence on the grain size, internal stress, dislocation density, texture, etc. [[40], [41], [42], [43], [44], [45], [46], [47]]. However, the results were controversial, and in some cases, grain refinement increased the corrosion rate because of the mechanical deformation and more grain boundaries.

Another approach to controlling the Mg degradation rate is the preparation of Mg metal matrix composite. Ideally, the reinforcement should be biocompatible and degradable. It should also enhance mechanical properties and bioactivity and decrease the corrosion rate. Many reinforcing particles were used, such as HA, β-tricalcium phosphate (β-TCP), bioactive glass, Ca-polyphosphate, fluorapatite, bredigite, Zn oxide, Mg oxide, Ti dioxide, graphene oxide, carbon, and polymers [[48], [49], [50], [51]]. In metal matrix composites, care should be taken to improve the interfacial bonding to avoid detachment of the reinforcement from the Mg matrix during the degradation. For instance, TiO_2_ enhanced Mg and graphene oxide bonding by forming TiC *in-situ* [50]. It is worth noting that nano-HA facilitates the formation of the calcium phosphate (CaP) protective layer and increases the corrosion resistance of Mg [52]. Moreover, spherical-shaped nano-HA significantly decreased the corrosion resistance compared to the needle-shaped HA [53]. The focus of the Mg metal matrix was on *in vitro* tests, and further *in vivo* studies, specifically biomechanical stability is still required.

Although the previous studies focused on controlling the corrosion rate of Mg alloys, here, the aim is to consider the possibility of incorporating bioactive agents to promote the bone healing rate. Therefore, this review aims to define the promising bioactive agents to be incorporated with Mg alloys for developing bioactive absorbable metals for high performance bone implants. It starts by reviewing the bone fracture healing mechanisms, the current progress in Mg alloys and bioactive coatings, and discusses the potential bioactive agents to accelerate bone regeneration.

## 2 Bone fracture healing process

The bone fracture healing process implicates dynamic interaction of different cell types, driven by multiple growth factors and signaling cascades and controlled by various physiological agents and cellular components [54,55]. There are two categories of bone fracture healing: primary (direct) healing and secondary (indirect) healing. Primary bone healing process is cortical remodeling without callus formation [56,57]. It requires a precise anatomical reduction of the fracture ends, no gap formation, and a stable fixation via open reduction and internal fixation surgery [58]. The healing occurs by direct remodeling of the lamellar bone, Haversian canals, and blood vessels once all the requirements are accomplished. Normally, it takes a few months to years for a complete recovery [59], but primary fracture healing is faster than the secondary healing process [60].

On the other hand, secondary bone healing involves responses in the periosteum and external soft tissues callus formation. Most bone fractures are healed by secondary healing via non-operative fracture treatment (immobilization) such as orthopedic cast or via certain operative treatments such as intramedullary nailing, and external or internal fixation of complicated comminuted fractures [61]. It implicates a combination of both intramembranous and endochondral ossification in the fracture healing sequence [62]. This category of fracture healing occurs at least by four complex phases: inflammatory, soft callus, hard callus, and remodeling phase [63] as summarized in Fig. 1b. Apart from that, fracture healing requires blood supplies, and revascularization is crucial for successful bone repair.

Upon bone fracture, the body protects the injury site by the acute inflammatory response of the soft tissue surrounding the fracture [64], leading to the formation of hematoma within the fracture gap [65]. Then, a fibrin-rich granulation tissue forms and within this tissue, endochondral formation occurs between the fracture ends, and external to periosteal sites. Various types of cells related to inflammation and immunity appear in the hematoma, including macrophages, neutrophils, and platelets that release several cytokines, such as platelet-derived growth factor, tumor necrosis factor-α (TNF-α), transforming growth factor-beta (TGF-β), interleukin-1 (IL-1), IL-6, IL-11 and IL-18 [66]. These cells release several biochemical factors to initiate cellular events [67]. After the resolution of the inflammation reaction, mesenchymal cells (MSCs) accumulate in the fractured injury site and form granulation tissue [68]. Bone MSCs are multipotent cells that play a role in bone regeneration and repair through their differentiation ability into various cells, including chondrocytes that form soft callus [69,70]. Subsequently, cartilaginous tissues are replaced via apoptosis by endochondral ossification that converts soft callus to hard callus (woven bone) [4,71]. Monocytes differentiate into osteoclasts that absorb the cartilage, while MSCs differentiate into osteoblasts that load lacunae resorption with new bone. This sequence leads to the formation of woven (hard) bones with trabecular structures. In the hard callus phase, the cartilage bone is taken over by the hard bone [56].

Finally, bone remodeling occurs where hard callus is mineralized, replaced with mineralized bone, and sculpted back to the bone's original shape and size with appropriate biomechanical competency [61,72]. This phase is coordinated by a balance of hard callus resorption by osteoclasts and lamellar bone deposition by osteoblast over a few months, and therefore, fully reinstate the biomechanical properties of a normal bone [73]. Biochemically, IL-1 and TNF-α show high expression, and at the same time, most members of the TGF-β family are diminished in expression during this phase [74,75]. In addition to that, selective bone morphogenetic protein (BMP) families such as BMP-2 are associated with high expression levels [76]. This process begins as early as 3–4 weeks, however in some cases, it may take years to complete and achieve a fully reformed bone structure. This phase also may occur faster in animal and younger patients. In addition, the process of fracture healing may also depend on several factors such as the patient's age, sex, health status, fracture severity, and location of the fracture [57,77].

The success of bone fracture healing is greatly influenced by biomechanical stability of the fixation system (implants) and revascularization of the fracture site [56]. The implant must maintain its mechanical stability for at least 3–4 months, where fracture callus transforms into new solid bone that recovers the inherent strength of most of the bone [78].

## 3 Absorbable magnesium alloys

Ideal biomaterials for bone fracture healing will have the following characteristics: (1) osteoconductivity to provide a place for blood vessel formation and bone ingrowth with a certain mechanical strength; (2) osteoinductivity to induce the expression of osteogenic proteins and stimulate surrounding stem cells to differentiate into chondrocytes or osteoblasts followed by mineralization and calcification until the new bone formation is achieved; and (3) osteogenesis to induce the differentiation of progenitor cells, osteoblasts, and bone progenitors into osteoblasts or their maturation [79]. The stem cells interact with biomaterials’ surface via adhesion, proliferation, and differentiation, indicating the importance of making the surface bioactive which can be modified by coating and other surface treatments.

**Fig. 2:**
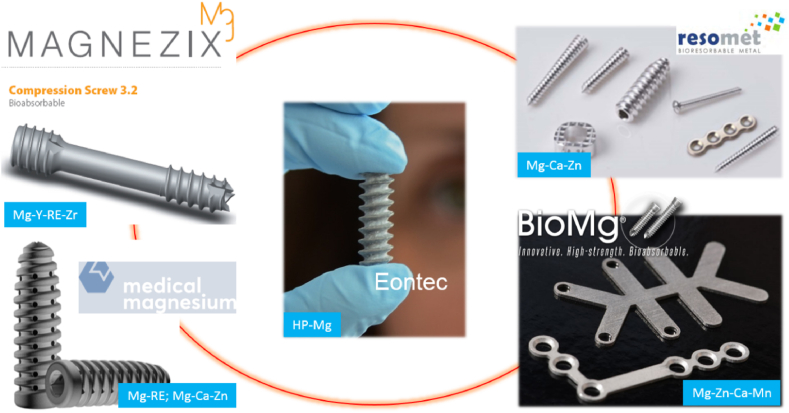
Examples of some commercial absorbable metal implants made of Mg and its alloys.

The nature of the bone fracture healing mechanism requires temporary mechanical support. Implants made of conventional corrosion-resistant alloys like titanium alloys, stainless steel 316L, and cobalt-chromium alloys will need to be retrieved via second surgery after healing is completed [15,80]. Implant retrieval and its associated cost and morbidity [81] and possible complication of bone re-fracture, infection and nerve damage [82,83] motivate developing absorbable metals. In almost twenty years of research in absorbable metals, Mg alloys have been viewed as the most suitable ones for bone implants than iron- and zinc-based alloys [2,84]. Fig. 2 provides examples of commercial absorbable metal implants made of Mg and its alloys.

Magnesium and its alloys are among the lightest structural metals with densities of 1.74 g/cm^3^ (pure Mg) and 1.75–1.85 g/cm^3^ (Mg alloys) that are very similar to that of human cortical bone, which is 1.75 g/cm^3^ [85]. Magnesium is an essential element in the construction of bone, soft tissues and acts as a cofactor for many enzymes [86,87]. Up to 30 g of it is restored in a healthy adult who is recommended to have a daily intake of up to 420 mg. Magnesium ion (Mg^2+^) is known to facilitate tissue healing, while its excess is excreted via urine and feces without causing any adverse effects [5,88]. The Mg ions are generated during degradation, and the resulting alkaline environment induces osteogenesis, thereby, increases osteoblastic mineral deposition and suppresses osteoclastic activities [[89], [90], [91]].

Magnesium and its alloys possess relatively low elastic moduli of about 45 GPa, close to the natural bone (3–20 GPa); therefore, a stress shielding between bone and implant will be less likely generated. However, pure Mg in as-cast condition has a very low strength, at just under 30 MPa, and a very fast degradation of 2.89 mm/year in 0.9% NaCl solution [92]. Its hexagonal close-packed crystal structure provides limited slip systems, limiting ductility and formability [93]. Purification, alloying, and severe plastic deformation are the common strategies to enhance mechanical properties and may provide increased degradation resistance at the same time [94,95]. Further control on the degradation behavior is mostly obtained through coating and surface engineering [9,96]. Table 1 shows tensile strength, ductility and degradation rate of Mg and its various alloys.

**Table 1:** Mechanical properties and degradation rate of Mg alloys.

Year	Alloy	Condition	UTS (MPa)	YS (MPa)	Elongation (%)	Immersion corrosion rate (mm/yr)	Medium	I_corr_ (μA/cm^2^)
2015 [97]	Mg–1Ca	Cast	105	39	–	–	–	–
2015 [98]	Mg–3Ca	Cast	–	–	–	–	SBF	929.3
		Rolled	–	–	–	–		74.2
2015	Mg-1.5 Sr	Homogenized + 24 h aged	81	40	–	–	–	–
	Mg–6Zn-0.5Sr		209	128	–	–	–	–
2015 [99]	Mg-0.5Ca	Cast and homogenized	–	–	–	2.79	SBF	–
	Mg–1Ca		–	–	–	0.66		–
	Mg-0.5Ca-0.5Zn		–	–	–	2.3		–
	Mg–1Ca–1Mn		–	–	–	2.82		–
	Mg–1Ca-0.5Zn-0.5Mn		–	–		2.09		–
2015 [100]	Mg–1Sn	Extruded	238.8	158.7	19.8	–	Hanks	5.15
2017 [101]	Mg-3Ge	Cast	50	150	10	–	Hanks	0.9
		Hot rolled	236	175	17.7	–		0.7
2018 [102]	Mg–1.8Zn–0.2Gd	Rolled	300	–	14	0.28	Hanks	–
2018 [103]	Mg–2Zn–0.46Y–0.5Nd	Extruded	268	159	12	0.2	SBF	–
2018 [104]	Mg–1Li–1Ca	Extruded	180	120	10	–	Hanks	6.49
2019 [105]	Mg–1Ca-0.5Zr	Heat treated	180–220		4–8	–	Hanks	3.85
2019 [106]	Mg-0.7Zn-0.6Ca	Hot rolled	–	–	–	0.12	α-MEM	5.13
2020 [107]	Mg–1Zn-2.9Y	ECAP	318	277	15	13	SBF	–
	Mg–2Zn-5.7Y	Extruded	430	364	4.6	2.3		–
2020 [108]	Pure Mg	HPT	167	117	29	–	–	–
	Mg–1Ca		315	229	1.6	–	–	–
	Mg–2Sr		253	166	2.6	–	–	–
2021 [109]	Mg–2Zn	Cast	80	150	3	0.4	SBF	–
		Hot rolled	260	223	3	0.2		–
2021 [110]	Mg–Zn–Ca–Mn	Homogenized	140	60	11.5	–	SBF	6.59
		Extruded	238	135	14.5	–		4.36
		Two-pass ECAPed	342	185	23.2	–		0.06

UTS: Ultimate tensile strength, YS: Yield strength, I_corr_: Corrosion current density.

HPT: High pressure torsion, ECAP: Equal channel angular pressing.

The wide variety of commercial and experimental Mg and its alloys can be grouped based on their alloy system: pure Mg, binary alloys, ternary alloys and many more. Highly (extra) pure Mg exhibits a low degradation rate due to elimination of iron-containing precipitates that usually form during casting and annealing in commercially pure Mg [111]. Previously, binary Mg alloys such as Mg–Ca, Mg–Zn, Mg–Sr, etc., have been studied, which showed high corrosion rate. Thermomechanical processes like rolling [98,102,109], extrusion [100,[103], [104], [105],^107^,^110^], high-pressure torsion (HPT) [108], and equal channel angular pressing (ECAP) [107,110] can refine the grains and suppress the formation of secondary phases along the grain boundaries, thus further improving the strength and degradation resistance of Mg alloys. In recent years, thermomechanical processing on ternary alloy systems attracted the scientific community to compromise between increasing the mechanical properties and decreasing the corrosion rate. Among all developed Mg and its alloys for bone implants, some have already been approved for clinical use, including high-purity (99.99%) Mg, Mg–Ca–Zn alloy, and Mg–Y-RE-Zr alloy [2].

Early trials to employ Mg as bone implants trace back to the early 1900s [112], where extreme degradation rate and poor refinement technology at that time hindered further exploration until the last decades. Now, the more advanced Mg alloys have been subjected to many *in vivo* studies revealing their good osteointegration and osteogenesis properties. For instance, Grünewalda et al. (2016) had shown that Mg ions released from high-purity Mg interference screws in anterior cruciate ligament (ACL) reconstruction of rabbit models resulted in an accumulation of BMP-2 and vascular endothelial growth factor that facilitated early phase tendon-bone healing [113]. Zhao et al. (2016) compared the same metal with titanium tendon graft healing screws in rabbits with ACL reconstruction and reported an excellent bone formation around Mg screws at an early stage of healing without bone tunnel widening [114]. In addition, reduced expression of matrix metalloproteinase-13 by the Mg screws resulted in an inhibitory effect on tendon graft degradation during the remodeling phase, providing a greater amount of collagen fibers in the tendon graft to be attached to the bone for better preclinical results [115]. Zhang et al. (2016) documented a gene-related calcitonin polypeptide-α mediated osteogenic differentiation promoted by Mg, showing the therapeutic potential of the metal in orthopedics [116]. A study by Xia et al. (2018) on mice femur (n = 10) showed a significant increase in cortical bone thickness around the Mg-3.5Li-0.5Ca alloy rods extracts that induced osteogenic differentiation of human bone marrow-derived mesenchymal stem cells (hBMMSCs) through the canonical Wnt/β-catenin pathway, without causing any adverse effects [117]. Some studies showed that Mg alloys can be used as Kirschner wires (K-wires) to stabilize bone fragmentations; however, the manufacturing processing of Mg wire, which is wire drawing, needs deep investigation as it influences the mechanical properties and the corrosion rate. Mg–2Ag [118], Mg-3Ge [101], and Mg–Zn–Mn [119] produced by hot extrusion, hot rolling, and cold extrusion, respectively, were promising candidates for K-wire applications. A recent study in 2021 compared Mg pins with stainless steel pins and K-wires for patella fracture fixation [120]. Magnesium groups showed higher mechanical strength and bone volume formation after 12 weeks of implantation in 32 female New Zealand White Rabbits. So, Mg pins can be a suitable candidate for the fixation of other surgeries which require K-wire.

**Fig. 3:**
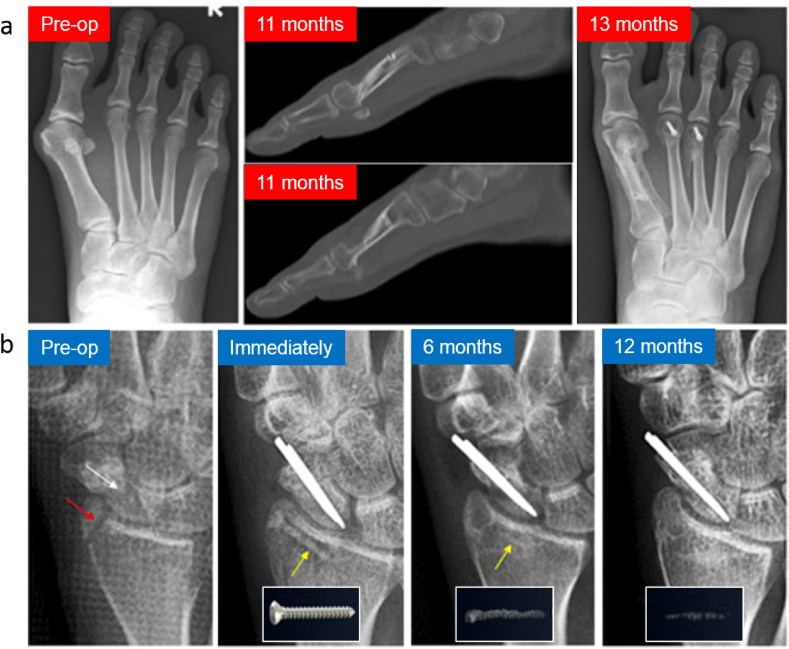
(a) X-rays images of a patient's left foot who received the MAGNEZIX compression screws made of Mg–Y-RE-Zr (MAGNEZIX®) alloy. Results were found that the screws were comparable with the treatment of hallux valgus abnormalities using titanium alloy screws [128] (b) X-ray images of the distal radius fracture and the scaphoid non-union before the surgical intervention (pre-op), implantation site immediately taken after the surgical procedures to fix the distal radius fracture with Mg alloy implant (Mg-5wt%Ca-1wt%Zn), 6-month follow-up, and 12-month post-operation where it shows the degradation of Mg alloy implant [115]. Adapted with permission from Elsevier.

In clinical trials, Mg implants have been tested to fix cases of bone fractures in Germany, China, Korea, Singapore, and Austria [115,[121], [122], [123], [124], [125]]. In 2013, Germany was the first country to report clinical treatment outcomes on orthopedic using Mg–Y-RE-Zr alloy (MAGNEZIX®) screws in hallux valgus abnormalities surgery [121].The same screws were then used in Ireland in 2015 to treat deformity in Madelung [126], followed by another case in Iran in 2016 to treat patients with a scaphoid fracture [127]. Subsequently, in 2017, MAGNEZIX® were used in a randomized clinical trial in German by Plaas et al. (2018) to treat patients with hallux valgus abnormalities [123]. Later, a prospective cohort study was accomplished in Singapore in 2018 using the same screw (Fig. 3a) [128]. In 2020, another case series by Plaas et al. (2020) in German used the same implant (MAGNEZIX®) to treat patients with hallux valgus abnormalities [124]. In China, Zhao et al. (2016) performed surgeries in patients suffering from osteonecrosis in the femoral head using specially designed high-purity Mg screws to fix vascularized bone flaps [114]. During the 12-month follow-up period, the Harris hip score and bone flap displacement using radiographic imaging showed significantly higher satisfactory therapeutic results in patients treated with Mg screw fixation. In the same year, a clinical trial in Korea by using Mg–Ca–Zn screws was performed to fix radius fractures (Fig. 3b). After six months post-surgery, the fracture was completely healed with no pain and no decrease in range motion reported, suggesting a normal healing rate in patients [115]. A complete replacement of Mg implants by new bone was observed within one year of implantation in 53 cases [129]. Most recently, Weldelstein et al. (2021) have executed a retrospective comparative study to compare the Mg screw vs. titanium screw vs. K-wire implants alloys. Table 2 summarizes the clinical trials of Mg alloys implants. In this table, eight studies of the clinical applications of Mg-based orthopedic implants were identified and analyzed. Most of the studies resulted from a single center/hospital, usually performed at 1, 4, 8, 12, 24 weeks to one year of observation following the implantation of the Mg implants. X-ray and low dose radiation Computed Tomography (CT) scan were performed to evaluate the bone healing process and determine the volume of formed H_2_ bubbles. Several indexes were used to measure the results. Overall, the results indicated no significant difference between the Mg alloy and titanium alloy groups, and normal healing occurred only in the Mg groups. Minor complications were noticed both in the control and Mg groups. These clinical data showed promising data for the future use of Mg implants. However, for a randomized control trial, a multicenter with a higher number of patients and longer observation time for clinical study are warranted in the future.

**Table 2:** List of clinical trials of Mg alloys implants.

Year	Type of study	No of patients (n) = (Mg alloy/control)	Observation time	Materials used	Location	Index measured	Result	Complication
2013 [121]	Randomized Control Trial	26 (13/13)	1–3d, 4–8d, 2wk, 6wk, 3mo, 6mo	MAGNEZIX vs titanium implant	Foot	AOFAS, ROM	No significant difference between groups	None
2015 [122]	Case series	19	3mo, 6mo,12mo	Pure Mg screw	Femoral Neck	HHS, CT	Satisfactory	Minor (1 case failed): avascular necrosis and non-union
2016 [114]	Randomized Control Trial	48 (23/25)	3mo, 6mo,12mo	Pure Mg screw vs without fixation	Femoral head	HHS, Xray, CT	HHS was significantly improved in Mg group	More in the none fixation group
2016 [115]	Case Series	53	1 wk, 2 wk, 1mo, 2mo, 3mo, 6 mo,12mo	Mg-5wt%Ca-1wt%Zn screw	Hand	Along with bone fusion assessment at 6 mo, passive range of motion, total active motion, hand grip power, DASH, and VAS	Normal healing rate	None
2017 [123]	Randomized Control Trial	26 (Full evaluation:8/6)	3y post-operative	MAGNEZIX vs titanium implant	Distal metatarsal	AOFAS, SF-36 questionnaire, FAAM, Pain-NRS, MRI	No significant difference between groups	None
2018 [128]	Prospective Cohort Study	93 (24/69)	Pre-operative, 3mo, 12mo post-operative	MAGNEZIX vs titanium implant	Distal metatarsal	AOFAS-HMI, VAS and all domains of the SF-36 questionnaire	No significant difference between groups	3 cases (12.5%) of superficial cellulitis and 1 case (4.2%) of neuropathic operative site pain
2020 [124]	Case series	70 (Full evaluation: 29/26)	6wk, 12 wk and 1 year	MAGNEZIX vs titanium implant	Foot	AOFAS, FAAM, NRS	Normal healing rate	Minor: pain during walking and running
2020 [130]	Retrospetive Case series	48 patients	12–53 months	Mg Screw vs Ti Screw	Ankle	AOFAS, The Kellgren–Lawrence (KL), CT	No difference between groups	None
2021 [125]	Retrospective Comparative Study	44 (16/16/16)	Minimum of 12 months	Mg Screw vs Ti Screw vs K wire	Foot	AOFAS, FFI, UCLA-A, VAS, Xray	No significant different between group in most of the index measure, however, Mg group significantly higher satisfactions	Minor but no significant different between groups

*AOFAS-American Orthopaedic Foot and Ankle Society analog scale for pain assessment, ROM- Range of Motion of The First Metatarsophalangeal Joint, HHS- Harris Hip Score, CT- Computerized Tomography DASH- Disabilities of The Arm, Shoulder and Hand, SF-36- Short Form 36 Health Survey Questionnaire, VAS- Visual Analog Scale, FAAM- Foot and Ankle Ability Measure, NRS- Numerical Rating Scale, FFI- Foot Function Index, University of California and Los Angeles Activity Score -UCLA-A.

## 4 Bioactive coatings

The high degradation rates of Mg alloy implants limit the time frame of their given mechanical support during bone fracture healing. Previous *in vivo* studies have shown that the rates of degradation of Mg alloy are too rapid to fulfill the bone repair requirement, although most findings have generally shown that *in vivo* degradation is slower than the measured *in vitro* degradation [131,132]. This feature of fast degradation is strongly connected to its electrochemical properties. With a low standard electrode potential of −2.37 V [133], Mg is extremely active. It has a high electronegativity from the perspective of electrochemical kinetics and is vulnerable to degradation in the physiological setting, which is rich in aggressive chloride ions. Not only does too-rapid deterioration of the Mg bone-implant lead to the premature loss of mechanical stability, but it also results in *in vivo* hydrogen accumulation, resulting in subcutaneous swelling and alkaline elevation at the implantation site [134,135]. Various coating strategies and surface treatments have been employed to postpone the start of degradation and add some surface bioactivities to accelerate bone healing [10,12].

Based on the requirements of metallic implants, different types of coating materials have been used to coat the implants. These coated materials can be divided into (1) bioinert coating and (2) bioactive coating. However, bioinert coating such as Al_2_O_3_, or ZrO only covers the thin layer or film on the implant's surfaces wherein unable to interact, respond to, or stimulate a chemical or bioactivity response with the surrounding tissues. Eventually, a layer of connective tissue is exposed in the interface, which is responsible for poor osseointegration. This order of events may cause the failure of the implant, and most of the cases may need a second surgery [136,137]. Therefore, there is a need for a coating surface that provides protection from corrosion and enhances the healing process. Lately, the usage of bioactive coating is gaining more attention as it helps an implant to mimic the natural properties of an organ, aims at enhancing the biomechanical anchorage, and induces osseointegration using either organic or inorganic bioactive materials [[138], [139], [140]]. These bioactive coatings i.e., HA, CaP or glass ceramics possess an ability for direct bonding with the living tissues, such as soft tissue or bone and establishing strong chemical and biological bonds. HA is frequently employed as bioactive material as it owns similar properties to a bone. An ideal bioactive coating shall provide sufficient support to promote and fasten the healing process of the bone within 3–6 months while also improving implant stability, enhancing soft tissue and peri-implant integration [[79], [80], [81]].

Another aspect of biocompatibility is the antibacterial ability to eliminate implant-associated bacterial infection. Pure Mg showed an antimicrobial effect according to the increase in pH by degradation. Other alloying elements such as Ag, Cu, Ga, and Zn can enhance Mg antibacterial activity, mechanical properties and decrease the corrosion rate [141,142]. Cu and Zn are also beneficial for bone regeneration and osteoblast activity, respectively [143,144]. However, there are concerns about their cytotoxicity. Therefore, microalloying or coating could be a better option. Metallic oxides, diamond-like carbon, and graphene has shown antibacterial as inorganic agents but those that decrease the corrosion rate of malloy are more favorable, e.g., SnO_2_ doped CaP [145], HA nanorods, ZnO nanorods [146], and nano-silica (SiO_2_)/graphene oxide (GO) [147].

### 4.1 Coating methods

**Fig. 4:**
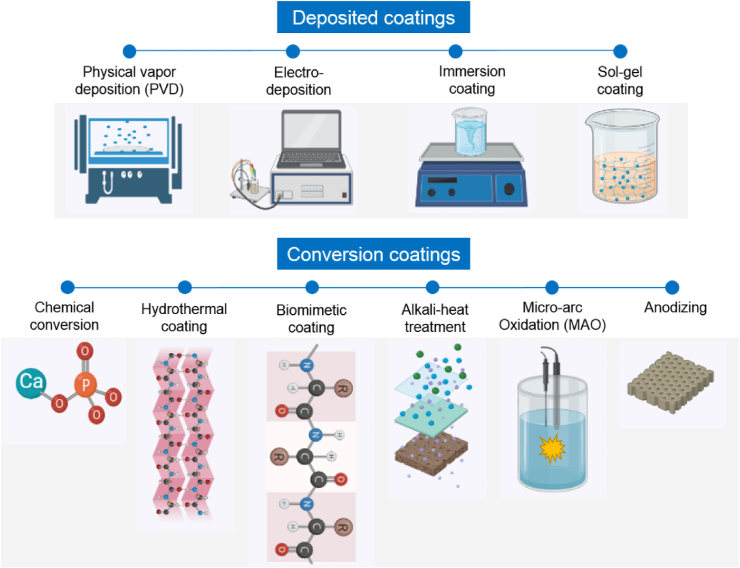
Schematic illustration of coating methods potentially suitable for Mg alloys.

The surface treatment of Mg alloys has been investigated with several methods but here we discuss those methods that provide a bioactive coating. Various types of bioactive inorganic and organic materials can be applied to coat Mg alloys via different techniques: (i) conversion coatings such as chemical conversion coatings, biomimetic coatings, micro arc oxidation (MAO), alkali-heat treated coatings, and hydrothermal, and (ii) deposited coatings such as physical vapor deposition (PVD), electrodeposition, immersion, and sol-gel. Fig. 4 illustrates the principles of each coating method.

#### 4.1.1 Conversion coatings

Chemical dissolution and precipitation are the basis of the chemical conversion coating. Calcium-phosphate is the most common chemical conversion coating in biomedical engineering, especially in orthopedics, according to the formation of biocompatible and osteoconductive HA layers [148]. The crystallinity of the layer is an important factor that is affected by the Ca/P ratio, and higher amounts of Ca^2+^ or PO_4_^3−^ can lead to the formation of amorphous phases such as Ca_3_(PO_4_)_2_, dicalcium phosphate dihydrate (DCPD; CaHPO_4_.2H_2_O), and CaHPO_4_.H_2_O [149,150]. The chemical conversion coating of CaP coating can increase the corrosion resistance of Mg alloys and enhance cell proliferation and bone growth on the interface of bone/implant [148,[151], [152], [153], [154]]. Another bioactive chemical conversion coating is fluoride conversion coating which forms an insoluble Mg fluoride (MgF_2_) layer. Ultrasonic fluoride treatment and double layer Mg/fluoride-Mg/phosphate treatments increased Mg alloys’ corrosion resistance, biocompatibility and bioactivity [155,156].

In biomimetic methods, different organic molecules such as ethylenediaminetetraacetic acid (EDTA) aids in formation of CaP with a noncovalent bonding mechanism. Cui et al. [157] used a biomimetic peptide (phosphophoryn, a bioactive extracellular matrix protein) to coat HA on AZ31B alloy that protected the substrate from corrosion and decreased hydrogen evolution. In 2019, a dense Mg(OH)_2_ layer formed on a porous MAO-coated Mg alloy by a biomimetic method, which increased the corrosion resistance of Mg alloy three times compared to the bare alloy [158]. Recently, silk was coated on the surface of anodized pure Mg by the biomimetic method [159]. Anodizing the substrate resulted in forming a rough surface and enhanced silk coating attachment which decreased the mass loss and improved cytocompatibility of the samples. MAO (also known as plasma electrolyte oxidation (PEO) or anodic spark deposition (ASD)) is a popular method to coat the surface of Mg alloys with ceramics. In this method, a high voltage is applied in an electrolyte, and the coating properties can be modified by adjusting potential, time, and frequency [160]. The coating layer is porous with an inner denser layer and an outer layer with higher porosities. The porosity helps higher cell/surface interaction; however, it can decrease the corrosion resistance by penetration of solution's ions. Thus, a post-MAO treatment can improve the corrosion resistance of the Mg alloys, such as hydrothermal treatment [161], electrophoretic deposition [162], polymer coatings (poly(lactic-co-glycolic acid) (PLGA)) [163], etc.

On the other hand, the incorporation of bioactive agents such as β-tricalcium phosphate (β-TCP) [164] and forsterite particles [165] can improve bioactivity. Alkali-heat treatment is a method to increase the surface roughness and it was applied to pure Mg mesh substrate to enhance CaP formation with a high osteointegration for guided bone regeneration [166]. A layered double hydroxide (LDH) is a typical form of hydrothermal coating. It consists of divalent and trivalent cations such as Mg^2+^ and Al^3+^ and interlayer anions like NO_3_^−^, PO_4_^3−^ that grow flake shape and vertically [167]. It can be performed on MAO coatings to improve the corrosion resistance [[168], [169], [170]]. Moreover, according to the structure and anion ex-change ability, LDH can carry drugs such as 5-fluorouracil (5-FU) for cancer treatments [171]. However, the temperature and time in LDH preparation should be precisely controlled, which makes the process difficult.

#### 4.1.2 Deposited coatings

Physical vapor deposition (PVD) is a technique that deposits a thin layer from a vaporized material. Standard PVD methods can form severe galvanic corrosion and need modifications [172]. Bakhsheshi-Rad et al. [147], developed a nano-silica/graphene oxide (SiO_2_)/GO coating with combination of PVD and deep coating on Mg alloy that increased corrosion resistance and exhibited antibacterial activity. Furthermore, microwave-assisted deposition helped deposit Ca-deficient HA coating and strontium-doped HA (Sr-HA) coating with improved mineralization ability [173,174].

Another method to deposit HA on the surface is electrodeposition. In this way, adjusting the Ca/P ratio and current mode applied to the solution can control the CaP composition [175]. To improve the adhesion of the layer to the substrate it is possible to apply it after the MAO process which can seal surface porosities and decreases the localized corrosion [176]. A pretreatment of Mg surface with a long-chain organic acid such as stearic acid was shown to modify the surface and make nucleation sites for CaP deposition by electrodeposition [177]. Recently, Rahman et al. (2021), prepared a bioactive hybrid coating on WE43 Mg alloy that consists of Mg(OH)_2_ inner layer by an anodization process, HA middle layer by electrodeposition process, and a silk fibroin outer layer by a spin coating process. The coating improved corrosion resistance, cell viability, attachment, and proliferation [180]. Besides CaP coating, electrodeposition can coat the surface with composites that release drugs and bioactive agents [178].

Immersion coating is a simple method widely applied to Mg alloys to decrease the corrosion rate and increase bioactivity by deposition of silanes [179], DNA [180], stearic acid [181], a natural polymer such as alginate [182], etc. However, it produces a thin layer, and the acidic solution used in this method can attack the Mg substrate. So, a combination of this method with other procedures like spin coating to prepare a multilayer coating is recommended. Sol-gel is based on the preparation of a solution with metal alkoxide. It is fabricated through a polymerization reaction, a hydrolysis step, and condensation which forms a gel film [183]. Sol-gel silica-based coating was useful to seal the porous structure after the MAO process on Mg alloy which prevented the diffusion of ions from the solution, consequently, improving corrosion resistance [184]. Table 3 summarizes the overview of bioactive coatings on Mg alloys.

**Table 3:** Overview of bioactive coating on Mg alloys.

Year	Alloy system	Corrosion behavior		Mechanical properties	Bioactive agent	Type	Coating technique	*In vitro*	*In vivo*
		I_corr_ (μA/cm^2^)	Corrosion rate (mm/yr)						
2020	AZ91D [185]	–	–	–	58S and 68S bio-glasses	Inorganic	Dip coating	Cell attachment and proliferation of mouse pre-myoblast auto-fluorescent cells were observed on both 58S and 68S coatings on AZ91D alloy	–
2020	Mg-Nd-Zn-Zr [186]	Uncoated = 0.52 Coated = 0.18	–	3-point bending load (N), *in vivo* Before implantation = 250 After 16 weeks: Uncoated = 40 Coated = 130	SrHPO_4_	Inorganic	Deposition	*-*	*In vivo* studies on femoral fracture rat model demonstrated that the formation of new bone induced and enhanced fracture healing in coated samples
2020	ZK60 [187]	Uncoated = 146 Coated = 1.36	–	–	Sr-doped CaP	Inorganic	Chemical immersion	–	Acceleration the process of new bone formation and better osseointegration was found around the coating than the alloy after four weeks of implantation in a rabbit model
2020	ZK60 [188]			–	Zn-doped nanowhisker HA	Inorganic	Hydrothermal treatment	Zn-HA coating promoted the adhesion and differentiation of rat bone marrow mesenchymal stem cells	–
2019	AZ31 [189]	–	a Uncoated = 5 Coated = 1	–	Sr-doped Zn–CaP	Inorganic	Chemical conversion	L929 cells showed higher cell viability of the Sr doped coatings compared to non-doped coatings	–
2019	AZ31, ZE41 [190]	–	–	–	Silane-TiO_2_/collagen	Inorganic		The silane-TiO_2_/collagen coating showed the improvement in cell response and viability of osteoblasts	–
2019	AZ91 [191]	–	–	–	HA	Inorganic	Radio frequency magnetron sputter deposition	Enhancement of bone marrow stromal cells (BMSCs) adhesion density in case of HA coating compared with the bare AZ91 substrate	–
2019	Mg [192]	–	–	Compressive strength (MPa) after 6 weeks Uncoated = 150 Coated = 250	Nano- and micro- HA	Inorganic	Transonic particle acceleration	Both nano- and micro-HA increased bone marrow derived mesenchymal stem cells (BMSCs) adhesion under indirect culture	–
2017	Mg/Ha [193]	–	–	–	Mg/HA scaffolds/recombinant human bone morphogenetic proteins-2 (rhBMP-2)	Inorganic	Immersion	MgHA/rhBMP-2 showed improved cell viability and proliferation and increased the expression of alkaline phosphatase (ALP), collagen type I and vascular endothelial growth factor (VEGF) protein.	*In vivo* results revealed effective osteogenesis and significant collagen I and VEGF mRNA expression at 12 weeks
2016	ZK60 [194]	Uncoated = 28.5 Coated = 0.26	–	–	Nano-HA	Inorganic	Hydrothermal treatment	Improvement in cytocompatibility properties of Murine fibroblast L-929 cells on the Mg alloy specimen	–
2014	AZ31 [195]	Uncoated = 74.2 Coated = 1.5	Uncoated = 1.7 Coated = 0.3	–	Si-doped calcium phosphate (CaP)	Inorganic	Electro-deposition	The coating showed a good cell growth and an enhanced cell proliferation and differentiation of MG63 osteoblast-like cells	–
2013	AM50 [196]	Uncoated = 103 Coated = 1.7	b Uncoated = 10 Coated = 2	Bending strength (MPa) As-rec = 300 After 20 days Uncoated = 200 Coated = 250	Polycaprolactone (PCL)/nano-HA composite	Inorganic	Dip coating	Level of osteoblastic differentiation activity was increased significantly with the incorporation of nano-HA into the PCL polymer matrix composite coatings on Mg implants	–
2012	Mg-Mn-Zn [197]	Uncoated = 32.5 Coated = 9.2	–	–	CaP	Inorganic	Immersion	L929 cells exhibit good adherence, growth, and proliferation characteristics on the coated Mg alloy	–
2011	Mg-Nd-Zn-Zr [198]	Uncoated = 38.3 Coated = 6.1	–	–	Calcium silicate and CaP composite	Inorganic	Chemical reaction	Good adhesion, high growth rates and proliferation of osteoblasts found on the coated Mg alloy	–
2011	Mg-Zn [199]	–	–	–	Fluoridated HA	Inorganic	Electrochemical method	Indirect cytotoxicity test on hBMSCs showed no toxicity at day 7	*In vivo* study on femoral condyle of adult New Zealand rabbits confirmed that the better interface contacts happened in the coated group 20 after one-month implantation
2011	Mg-Zn-Ca [200]	–	Uncoated = 1 Coated = 0.8	–	Ca-deficient HA	Inorganic	Pulse electrodeposition	–	Acceleration the process of new bone formation in adult rabbit around the coated Mg implants after 24 weeks implantation
2010	Mg-Zn [201]	–	–	–	Fluoridated HA	Inorganic	Electrochemical method	Good cellular proliferation and differentiation of hBMSCs were observed in case of bioactive fluoridated HA coating	–
2020	Mg-Zn [202]	Uncoated = 42.6 Coated = 1.8	–	–	Dopamine/gelatin/rhBMP-2– coated β-TCP	Organic	Powder processing	Extracts from the dopamine/gelatin/rhBMP-2-coated β-TCP/Mg–Zn composite facilitated cell proliferation and significantly enhanced the osteogenic differentiation of Sprague-Dawley rat bone marrow-derived mesenchymal stem cells *in vitro*.	*In-vivo* test on New Zealand rabbit showed strong promotion of new bone formation, matched composite degradation and bone regeneration rates
2019	AZ31B [203]	Uncoated = 33.3 Coated = 0.9	–	–	BMP-2	Organic	Micro-arc coating, and layer-by-layer	–	BMP-2-loaded groups exhibited better biodegradation rate and osseointegration than the control group in 2 weeks of implantation. After four weeks, the group with 50 ng/mL of BMP-2 showed the lowest biodegradation rate of all the BMP-2-loaded groups
2019	AZ31B [204]	Uncoated = 88.6 Coated = 0.75	–	–	Chitosan/heparinized graphene oxide	Organic	Layer-by-layer method	The multilayer coating promoted the adhesion and proliferation of endothelial cells	–
2019	Mg-Gd [205]	–	–	–	Chitosan-Mg composite	Organic	Dip coating	–	Higher amounts of new bone in rabbits were formed for the chitosan coated samples
2014	AZ31D [206]	Uncoated = 625 Coated = 70	–	–	Bioactive carboxymethyl chitosan	Organic	Immersion	Cytotoxicity test and cell morphology analysis confirmed that adhesion and proliferation of osteoblasts on the modified alloy surface were improved	–
2019	Mg-Sr [207]	–	–	–	Zoledronic acid associated with CaP	Drug	Bilayer coating	The bilayer coated Mg–Sr alloy enhanced proliferation, osteogenic differentiation, and mineralization of pre-osteoblasts, however, induced apoptosis and inhibited osteoclast differentiation, which promoted the balance of bone remodeling process	–
2019	WE43 [208]	Uncoated = 6.05 Coated = 2.15	–	–	Simvastatin, gelatin nanospheres/chitosan (GNs/CTS) composite	Drug	Electrophoretic deposition	Simvastatin-loaded GNs/CTS composite coatings were able to enhance the degradation resistance of WE43 substrate and promote osteogenic activity	–

a Converted from weight loss (mg/cm^2^/h) 96 h: Uncoated = 2.5, Coated = 0.6.

b Converted from weight loss (mg/cm^2^) 20 days: Uncoated = 5, Coated = 1.

### 4.2 Inorganic coating

Inorganic materials such as HA, CaP, and fluoride have been recognized as bone substitute materials due to their similar chemical composition to natural bone. They are conducive to bone tissue growth at the bone-implant interface [209]. In fact, HA (Ca_10_(P0_4_)_6_(OH)_2_) has been used as bone filler and coating on metal prostheses [210]. Surmeneva et al. (2019), through an *in vitro* study, showed that hBMSCs were found to attach on HA-coated AZ91 alloy, demonstrating spindle-like shape morphology typical for proliferating BMSCs [191]. Compared to uncoated Mg–Zn–Ca alloy, the Ca-deficient-HA coated one showed a remarkable proliferation of osteoblasts and more new bone formation in the first eight weeks of implantation in New Zealand White rabbits which then matured within 18 weeks post-implantation [200]. The enhanced activity of the osteoblasts around the coated implant might be due to the osteoconductive nature of bone-like apatite chemistry of the coating materials (trace of Na^+^, Mg^2+^, CO_3_^2−^, Ca^2+^ and PO_4_^3−^) and the reduced degradation rate that allowed a balance rate between ions release by the implant and their absorption by the tissue during the bone formation process [211]. The CaP-based coating on Mg–Mn–Zn alloys was found to induce cell attachment, growth, and proliferation of L929 cells, owing to the provision of Ca^2+^ ions that assisted the absorbance of proteins like fibronectin and vimentin for cell adhesion and spreading [197].

**Fig. 5:**
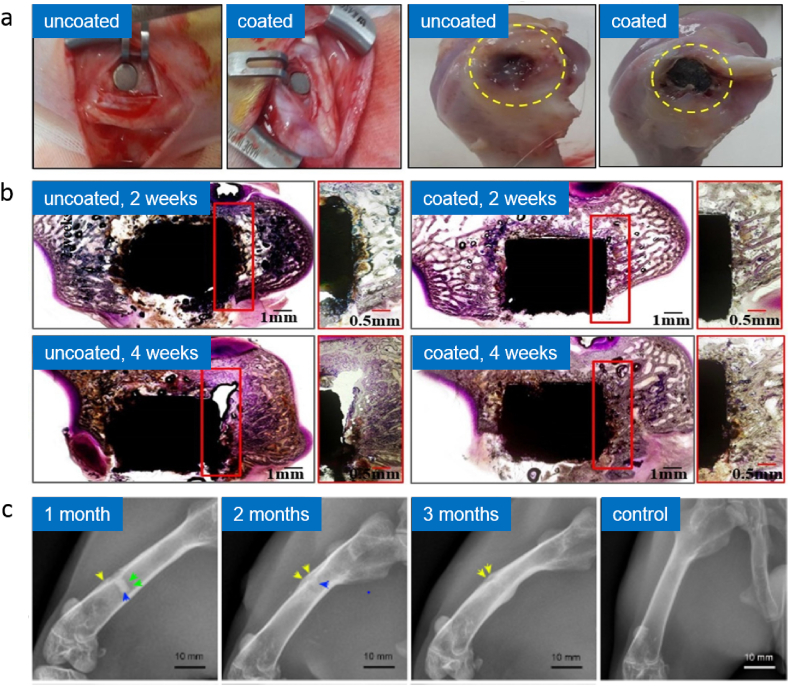
*In vivo* study of Sr-doped CaP coated ZK60 alloy specimen in rabbits, showing (a) photographs of implantation and histological sectioning of uncoated and Sr-doped CaP coated ZK60 alloy specimen in rabbits, and (b) histological micrograph after 2 and 4 weeks of implantation [187]; (c) *in vivo* study of dopamine/gelatin/rhBMP-2–coated β-TCP Mg–Zn alloy showing radiographs of the implantation site of the rabbit's femur at anteroposterior view for 1, 2 and 3 months post-implantation [202]. Adapted with permission from Elsevier.

A Sr-doped CaP coating on ZK60 alloy was found to promote adhesion, proliferation, and expression of osteogenic markers of MC3T3-E1 cells and to enhance bone formation and osteointegration for four weeks post-implantation in the rabbit model compared to the uncoated alloy (Fig. 5a–b) [187]. Ca^2+^ ion was known to favor cell activity [212], while the addition of strontium facilitated cell and protein binding via different surface cell receptors and resulted in an active environment for enhancing cell growth. A Si-doped CaP coating was performed on AZ31 alloy and preliminary cytocompatibility evaluation of the coating using osteoblasts showed that silicon ions play an important role in the nucleation and growth of apatite and thus influence the biological metabolism of osteoblastic cells in the bone formation process [195]. Zinc is an important trace element that is found in human bones, which plays vital roles in biological functions, such as DNA synthesis, enzyme activity, nucleic acid metabolism, biomineralization, and hormonal activity [213]. The results of Zn-doped HA coating on the ZK60 alloy plate showed that the HA-Zn has a better effect on promoting the osteogenic differentiation of BMSCs than those of the other groups. A possible explanation for these results may be that an appropriate zinc concentration is beneficial to the osteogenic differentiation of BMSCs. After implantation, adhesion and spreading of BMSCs on the implant's surface is the first step in osseointegration [188].

In addition, fluoride is essential for normal dental and skeletal growth and may promote the proliferation of osteoblasts and increase new mineral deposits in cancellous bones [213,214] Liu et al. (2018) stated that fluoride treatment on Mg alloys replaces the original oxide film with a thin and more homogeneous MgF_2_ layer that was dense, less soluble in water, and nontoxic to organisms [215]. An MgF_2_ coating was found to slow in an *in vivo* degradation of LAE442 alloy without observably elevating fluoride concentrations in the adjacent bone [216]. Sun et al. (2016) implanted fluorine-coated AZ31B alloy screws in rabbits' mandibular and femur, resulting in up-regulated expressions of collagen type I and BMP-2 and enhanced osteogenic activity [217]. The enhancement of osseointegration was also observed on Mg–F-coated Mg–Ca implants as the new bone formation was observed at the edges of the implant and an endosteal and periosteal remodeling [218]. Another study on fluoridated HA coating on Mg–Zn alloys implanted in the femoral condyle of adult New Zealand rabbits showed an enhancement of interfacial bioactivity mainly due to a quick cell's differentiation as the result of more direct implant-tissue contacts on the coated implants compared to the non-coated ones [199]. Doping of Mg in CaP/sodium alginate composite coating provided a higher hardness to the coating that can improve the scratch resistance of the coating [219] while alginate is a biocompatible substrate for drug delivery.

### 4.3 Organic coating

Organic materials such as chitosan, polydopamine, and bone morphogenetic protein-2 (BMP-2) are among the bioactive materials applied for coating on Mg. Chitosan (1–4,2-amino-2-deoxy-beta-D-glucan), a de-acetylated derivative of chitin found primarily in the arthropods exoskeletons, is considered a type of biopolymer that possesses osteoconductive properties [220]. Magnesium-chitosan was found to have conducive characteristics to protein adsorption and can provide a good platform for cell adhesion and proliferation [221]. Chitosan films potentiated the differentiation of osteoprogenitor cells, facilitated bone formation and inhibited fibroblast proliferation [222]. Guo et al. (2019) reported that dip-coated chitosan on Mg alloy functioned as a biodegradable barrier membrane in guided bone regeneration [205]. The addition of collagen into CaP coating vastly improved osteoblasts’ viability as it provides suitable conditions for cell attachment and proliferation [223].

Polydopamine, a final oxidation product of dopamine or other catecholamines, has become a versatile coating material that can cover the different surfaces with a conformal layer of adjustable thickness from a few to about 100 nm [224]. This biomimicry material has strong adhesion properties and high-cell affinity [225,226]. A coating strategy developed by Jiang et al. (2017) employed polydopamine mediated assembly of HA-coated alkaline-treated nanoparticles and immersion of BMP-2 onto the surface of AZ31 alloys resulting in significant cell adhesion and proliferation of BMSCs in rats and enhanced osteoinductivity and osseointegration in the New Zealand rabbit model [227]. Guo et al. (2019) developed a multifunctional composite coating composed of polydopamine, dicalcium phosphate dihydrate, and collagen on AZ60 alloy by a two-step chemical method that has a similar composition to natural bone a favorable interface for MC3T3-E1 cell viability and adhesion [228]. Peng et al. (2020) also observed an enhanced osteogenic differentiation ability of MC3T3-E1 on Zn-contained polydopamine film of AZ31 alloy, and an enhanced osteogenesis and osteointegration 8 weeks post-implantation in Sprague-Dawley rats [229].

Bone morphogenetic proteins are primarily shown to induce ectopic bone growth, enhance chondrogenesis and osteogenesis [230,231]. Katiella et al. (2016) showed that BMP-2 coated Mg alloy promotes the expression of bone growth factors in New Zealand rabbits, thus delaying femoral head necrosis and improving its reparation [232]. A polydopamine mediated HA coating via alkaline-treated nanoparticle immerse with BMP-2 on AZ31 alloys revealed that immobilization of HA nanoparticles and BMP-2 promotes cell adhesion and proliferation. It also indicates synergistic effects in inducing new bone formation during implantation tests in New Zealand rabbits without an obvious inflammatory response [227]. Kim et al. (2019) deposited various concentrations of BMP-2 in the carrier MgO and Mg(OH)_2_ layer of AZ31B alloy via a micro-arc coating. There results showed significant proliferation and differentiation of osteoblast cells that were promoted by the continuous release of 50 ng/mL of BMP-2 after four weeks, thus enhancing new bone formation and a stable bone growth [203]. Their further study proved that placement of BMP-2/HA within a cannulated Mg screw enhanced the bone formation ability, higher osteointegration between implants and host femurs at 12 weeks, which replaced the gas void around the implants in the New Zealand rabbit model, indicating its potential to limit the complications of hydrogen gas accumulation [233]. A recent study by Liu et al. (2020) indicated that extracts of dopamine/gelatin/rhBMP-2-coated β-TCP/Mg–Zn composite facilitated cell proliferation and significantly enhanced an *in vitro* osteogenic differentiation of BMSC. While in an *in vivo* experiment on rabbit femoral shaft, the coated composites improved early osteoinductivity with a strong promotion of new bone formation, matching composite degradation with bone regeneration rates, and complete hydrogen gas absorption (Fig. 5c) [202].

### 4.4 Drug coating

**Fig. 6:**
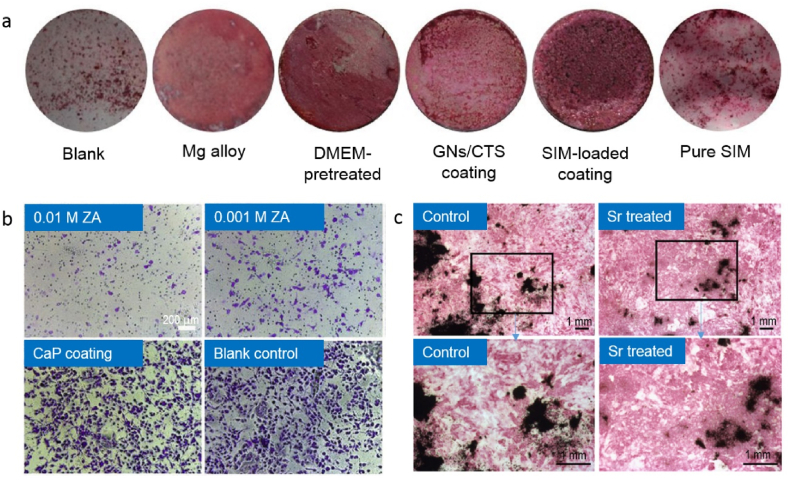
Studies on coating of Mg alloys with simvastatin (SIM), zoledronic acid (ZA), and strontium (Sr): (a) ARS staining shows matrix mineralization for MC3T3-E1 cells after 18 days with SIM-loaded sample showed the highest mineralization nodules [208], (b) staining of migrated pre-osteoclasts for different ZA coatings, CaP coating and blank sample, showing ZA coating effectively decreased the pre-osteoclast migration [240], (c) ALP staining of primary fetal mouse calvaria cells proved bone nodules formation for the cells treated with Sr [241].

Equally appealing in enhancing bioactivity of Mg bone implants is by coating with osteoinductive drugs, such as simvastatin and zoledronic acid. The drugs could be loaded onto the surface coating, placed in a cannulated hollow, or encapsulated into a scaffold. Simvastatin is one of the lipid-lowering drugs prescribed in clinics [234,235]. However, this drug has been used to explore its effects on osteogenesis in recent years [236,237]. Simvastatin was found to promote the formation of new bone [238], differentiation of osteoblasts and mineralization of MC3T3-E1 extracellular matrix [239]. Local delivery can avoid the severe side effect from systemic usage of this drug, such as in a coating system [208]. Qi et al. (2019) showed the potential of simvastatin-loaded gelatin nanospheres/chitosan composite coating on WE43 alloy, fabricated by electrophoretic deposition, on inducing osteogenic differentiation of MC3T3-E1 cells by maintaining its pharmacological activity through up-regulating the expressions of osteogenic genes and related proteins (COL-1, OCN), promoting alkaline phosphatase activity and enhancing extracellular matrix mineralization [208]. Li et al. (2018) observed ZA's potential for incorporating Mg–Sr alloy as bone substitutes for effective therapy to reduce osteolysis [240]. Bonnelye et al. (2008) summarized studies that showed Sr^2+^ leads to an increase in the bone-to-implant contact, peri-implant bone volume, and push-out force [241] (Fig. 6).

## 5 Activating magnesium with bioactive agents

Modifying the surface of metallic implants with a thin layer of bioactive materials attached through covalent bonding represents an attractive strategy to improve the implant's bioactivities [242]. Bioactive coatings have been rapidly developed by incorporating various polymers, organic and inorganic materials [243,244]. These coatings can be applied onto Mg implants by using sol-gel, electrophoretic and electrochemical deposition, as shown in Table 3. The coating offers simplicity, low cost, low process energy, and precise control of coating parameters result in highly uniform thin films and excellent penetration, and the capability to form complex shapes [245,246].

**Fig. 7:**
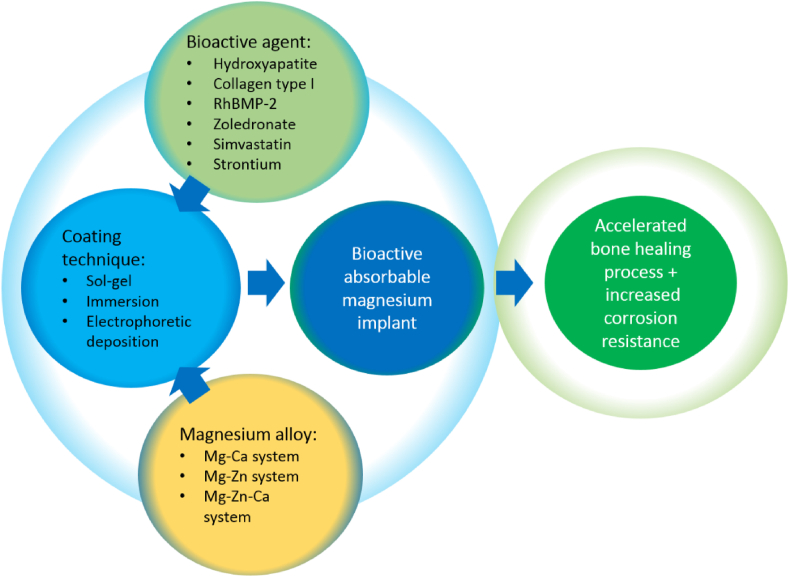
Proposed strategy for achieving bioactive absorbable Mg implants that promotes accelerated bone healing process and increased corrosion resistance.

A well-developed bioactive coating on Mg implant serves as a biofunctional layer that provides improved biocompatibility and degradation resistance. Thus, a bioactive coating platform, proposed in Fig. 7, that promotes both osseointegrations that enhance the healing process and increases corrosion resistance should be considered one valid strategy to deal with the rapid degradation issue of Mg implants. A well-suited bioactive agent with a coating technique applied onto a selected Mg alloy could result in a high-performance absorbable Mg bone implant for orthopedic applications.

### 5.1 Potential bone regeneration enhancing elements

The primary purpose of alloying Mg is to improve structural properties such as strength and ductility. Some specific alloying elements can also have a role in bone regeneration, mainly Ca, Zn, Cu, and Mn. Calcium is the main component of human bones, and teeth are present as HA crystals that play an important role in maintaining skeletal framework [247]. Surface functionalization with HA coatings has been proven to improve metal implants' osteoconductive and osteoinductive performance [242,248,249]. Zinc shows a capacity to stimulate osteoblast bone formation, increase alkaline phosphatase activity, and inhibit osteoclast differentiation [[250], [251], [252]]. Copper ion (Cu^2+^) enhances the activity and proliferation of osteoblasts, promotes osteostimulation, and exhibits antibacterial effect [[253], [254], [255]]. Manganese is associated with the maintenance of the bone structure and regulating bone metabolism. An accelerated bone fracture healing was observed in a rat model after being administered with local treatment of Mn^2+^ [256,257]. These elements can be carefully chosen by considering their level of toxicity in the human body and their metallurgical role in forming an alloy's microstructure and dictating mechanical properties and degradation behavior.

### 5.2 Potential bioactive agents

Among all the bioactive coatings, six highly potential coatings are proposed for further use on Mg implants: HA, collagen type I, RhBMP-2, simvastatin, zoledronate, and strontium. These coatings might be combined or may work as a single agent in accelerating bone fracture healing. Table 4 shows the most potential bioactive coating system and its expected clinical outcomes to be further explored.

**Table 4:** Potential bioactive coating system for high-performance absorbable Mg bone implants.

Bioactive agents	Advantages	Disadvantages
HA	Easy to handle, good bioactivity and biocompatibility, hydrophilicity, similar to inorganic components, good osteoconductivity and good potential osteoinductivity [258] Enhancing implant fixation within 9–10 weeks	Very brittle, high stiffness, low flexibility [259]
Collagen type I	High biocompatibility, enhanced cellular interaction, hydrophilicity, enhanced cellular interaction, providing secondary stability to the implant and osteoconductivity over a period of 4–12 weeks [260]	Rapid degradation rate, low mechanical strength [261]
Recombinant human BMP-2	Accelerating and enhancing early osteoinductivity and osseointegration with a strong promotion of new bone formation in less than 12 weeks [262]	Side effects i.e., inflammatory reaction, radiculopathy, ectopic bone formation, osteoclast formation, urogenital complication, and wound complications [263]
Simvastatin	Accelerating bone formation at implant surface and enhancing osseointegration [264], low risk of drug toxicity and side effects [265]	Difficult in the delivery system and dose dependent effect on bone healing [265]
Zoledronate	Reducing osteoclastic activity, increasing the mechanical strength of a healing fracture by retaining new-formed callus volume [266], enhance pin fixation [267], shorten the fusion less than 24 weeks [268]	Side effects i.e., gastrointestinal irritation, osteonecrosis of jaw and impairment of renal function in systemic use [269]
Strontium	Suppressing osteoclast activity, Enhancing bone formation and mechanical strength [241]	High dose of Sr^2+^ results the occurrence of hypocalcaemia, caused by an increase in renal excretion of Ca^2+^ ions [270]

#### 5.2.1 Calcium phosphate and hydroxyapatite

Calcium phosphate ceramics such as tricalcium phosphate and HA are excellent candidates for bioactive coatings. Owing to the properties of bone components, they have intrinsic bioactivity and biocompatibility for orthopedic applications [271]. Augmenting HA with a polymer matrix tends to improve its mechanical strength and bone-bonding ability. They have also been used as ion delivery vehicles within bone regeneration. Several ions such as Ca^2+^, PO_4_^3−^, F^−^, and Sr^2+^ are capable of inducing osteoblast precursor differentiation through growth factor signaling pathways, or to stimulate other processes in support of bone tissue growth [271]. The inorganic HA phase of bone tissue contains approximately 99% of the Ca in the body, acting as a storage reservoir for the mineral [272]. Calcium signaling also has a role in the stimulation of bone synthesis pathways in osteoblasts through interaction with the calmodulin protein and activation of extracellular-signal-regulated kinase 1/2 (ERK1/2) during mechanical stimulation and associated increased fluid shear in the bone [273]. Downstream effects of Ca signaling include activation of the phosphoinositide 3-kinase/protein kinase B (PI3K/Akt) pathways, which supports the continued survival of osteoblasts [274]. Approximately 85% of phosphorus complexed with Ca, in the form of HA, is found in soft tissue and extracellular fluid of the human body. In osteoblasts and pre-osteoblasts, phosphate participates in bone formation by regulating the proliferation (partly via an increase in IGF-I), differentiation, and mineralization of the cells via ERK1/2 signaling pathway, and apoptosis through decreasing the mitochondrial transmembrane potential. This pathway is accentuated by Ca^2+^ ions [275].

#### 5.2.2 RhBMP-2

Growth factors and hormones such as bone morphogenetic proteins (BMPs), fibroblast growth factor (FGF), insulin-like growth factors (IGFs), platelet-derived growth factor (PDGF), transforming growth factor-beta (TGF-beta), and vascular endothelial growth factor (VEGF) are widely recognized to play an important role in bone repair. Previous research on bone fracture healing focused on the local application of substances on the implant surface [[276], [277], [278]], or directly in the implantation site, able to accelerate the osseointegration process. Hormones [[279], [280], [281]], growth factors [282,283], and BMP proteins [284,285] are being used to stimulate bone growth. The role of RhBMP-2 in the regulation of fracture healing has been established; however, the molecular mechanisms of action are still being explored [286,287]. Deng et al. (2017) revealed that Mg-HA scaffold combined with rhBMP-2 improved cell viability and proliferation of MG63 cells and could increase the expression of alkaline phosphatase, collagen I, and VEGF protein compared with pure HA on HUVEC cells. The combination also improved the calvarial defect repair effect in the goat model by showing the most effective bone formation outcome [193]. In the context to improve bone formation and fracture healing processes, the combination of the rhBMP-2 with absorbable collagen may be a suitable and safer alternative for bone repair purposes [288]. However, in 2008, the FDA received 38 complaints of problems related to the use of BMP-2 in anterior cervical surgery that was performed “off-label.” A review by James et al. (2016) have listed all the clinical and preclinical side effects of BMP-2, including inflammatory complications, radiculopathy, ectopic bone, osteoclast activation and osteolysis, urogenital events, and wound complications [263]. Due to these side effects, more research into rhBMP-2 is required to improve long-term results, investigating the alternative Mg alloying systems, scaffolds, or biocomposites that could be used in conjunction with rhBMP-2, and assessing the cost–benefit of rhBMP-2 to the healthcare system.

#### 5.2.3 Collagen type 1

A wide variety of natural polymers, i.e., collagen, chitosan, glycosaminoglycans, synthetic polymers (polyglycolic acid (PGA), polylactic acid (PLA), copolymers of lactic and glycolic acids (PLGA), polyanhydrides (polyorthoesters), polyurethanes, silicones) and others are used in the production of bioactive materials for biomedical applications. These polymers allowed an increase in bony integration behavior. Among all these polymers, collagen type 1 appears to be distinctive from the others due to its resemblances with naturally occurring collagen in our body [289]. It helps to enhance tissues regenerations such as bone, tendon, ligament, skin, vascular and connective tissues [290]. Due to the coherency with the biological property with that of native collagen that already exists in our body system, it may also function as cell scaffolds for tissue engineering applications. Mushahary et al. (2014) developed a collagen type I coating on Mg alloy They demonstrated that the collagen type 1 coating improved the surface hydrophobicity and energy of alloys and accelerated the protein binding capacity onto the alloy surface, resulting in better osteoblast activity. Furthermore, it improved the implant stability and osseointegration rate after only a month of implantation, as demonstrated by histology, immunohistochemistry, and radiology. Therefore, it is expected that the composite coating containing CaP and collagen can better simulate the composition of bone [260], improving the osseointegration between the implant and bone in less than 12 weeks. Nevertheless, collagen type 1 has some limitations, such as low mechanical strength that can be improved by modifying collagen cross-linking [261]. Despite their advantageous biological properties, natural polymers showed a rapid degradation rate related to low mechanical strength. To overcome those limitations, natural polymers are usually combined with bioactive materials (i.e., bioceramics) or mechanically strong materials (i.e., synthetic polymers or metals) [270].

#### 5.2.4 Simvastatin

Simvastatin has been studied extensively for its osteopromotive properties since the 1990s. Simvastatin is a member of the statin family, which are molecular analogues of HMG-CoA (3-hydroxy-3-methylglutaryl-coenzyme A), have been generally used to lower blood cholesterol, but recent studies have also reported to decrease rate of bone resorption and increased bone mineral density in statin users. Statins could reversibly inhibit HMG-CoA reductase by binding to the enzyme's active site and blocking the catalyst's substrate–product transition state [291]. The major mechanisms of simvastatin action on bone include promoting osteogenesis, inhibiting apoptosis in osteoblast; and suppressing osteoclastic differentiation and activity. This drug increases osteogenesis by enhancing mesenchymal cells differentiation into osteoblasts, upregulating bone morphogenetic protein-2 (BMP-2) and downregulating osteoblast apoptosis [265]. Simvastatin cannot be metabolized by the liver, hence, reduce the risk of drug toxicity and others side effects [265]. According to a review published in 2019, the local application of statins on animal models promotes the healing of critical bone size defects due to its apparent osteogenic and angiogenic effects [292]. The delivery of simvastatin to bone defects using methylcellulose gel [293,294], gelatin hydrogel [295], collagen sponge [296], or gelatin sponge [297] has shown an enhanced bone healing by radiological and histological assessment. Immunohistochemistry confirmed increased expression of BMP-2 at the site of simvastatin delivery [[294], [295], [296]]. However, studies using rats have demonstrated that high doses of simvastatin (0.5–2.2 mg per site) may induce an inflammatory response [298,299] or even impair bone healing (30 mg/kg) [300], which may hinder the future clinical use of simvastatin. Therefore, there is a need for a controlled delivery system that could release simvastatin in an appropriate dose range. After all, encouraging results have been achieved by delivering low-dose simvastatin (250 lg) on a fracture site. In that study, Fukui et al. (2012) performed a femoral fracture in a rat such that a non-union persisted eight weeks later, which was then treated by a gelatin hydrogel releasing a low dose of simvastatin. The results revealed a significant improvement in fracture healing, with 71% of the treatment group showing a union, in comparison to the 7% of the control group with hydrogel alone [301].

#### 5.2.5 Zoledronate

In the past 30 years, bisphosphonate use is mainly connected with clinical use of stainless steel or titanium alloy implants, and their efficacy has been proven both *in vitro* and *in vivo* [302]. In animal fracture tests, bisphosphonates improved callus size and power. Bisphosphonates reduced the recovery period by 12 days in a human non-randomized pilot trial of high tibial osteotomies in knee osteoarthritis using the hemicallotasis (HCO) procedure [303]. Zoledronic acid (ZA), also known as zoledronate, is a long-acting bisphosphonate, could be given as an annual intravenous infusion to increase callus volume significantly. Kates and Ackert-Bickel (2016) claimed that osteoclasts would pick up zoledronate when it is resorbed and released in the acidic lacuna of the shattered bone. Farnesyl pyrophosphate synthase (FPPS), a crucial enzyme in the mevalonate pathway, is inhibited by zoledronate. This reaction causes cytoskeletal alterations in the osteoclast, which decrease the osteoclast's activity and/or cause apoptosis in these cells [304]. However, considering the undesirable side effects such as gastrointestinal irritation, osteonecrosis of the jaw, and impairment of renal function in systemic use [269], local administration of this drug directly targeting the location of osteoclast action, seems to be more effective. Li et al. (2016) observed ZA's potential for incorporating Mg–Sr alloy as bone substitutes for effective therapy to reduce osteolysis [207]. They prepared a novel bilayer coating on Mg–Sr alloy by the deposition of CaP and ZA and found that local delivery of ZA could enhance the osteogenic proliferation and differentiation as well as the mineralization of pre-osteoblasts MC3T3-E1; however, it induced apoptosis and inhibited osteoclast differentiation.

#### 5.2.6 Strontium

Strontium has also widely been used to enrich biomaterials such as various kinds of CaP, bioactive glass, bone cement, and metallic implant. Strontium is structurally, physically, and chemically similar to Ca and, thus, has been studied extensively in bone regeneration. Strontium is a strong bone-seeking trace element, of which approximately 98% is localized in human bone tissue [305]. The introduction of elements such as Mg^2+^, Zn^2+^, Sr^2+^, Si^4+^, F^−^ within HA, help to improve its chemical and biological properties such as the degree of structural order (i.e., crystallinity), solubility in chemical solvents, surface charge, and dissolution rate under simulated physiological conditions [306]. Among the various cations that can replace Ca in the HA lattice, strontium has been gaining interest due to its physical and chemical similarity to Ca. Strontium interaction mechanism with bone tissue also the same manner that takes place physiologically with the participation of Ca. Both elements accumulate in plasma and extracellular fluids, soft tissues, and the skeleton [307]. The presence of Sr^2+^ in these structures enhances the proliferation and osteogenic differentiation of osteoblastic cells and inhibits an *in vitro* osteoclast activity. However, *in vivo*, Sr^2+^ incorporation promotes bone formation, remodeling, and osseointegration. Bonnelye et al. (2008) summarized studies that showed Sr^2+^ leads to an increase in the bone-to-implant contact, peri-implant bone volume, and push-out force [241]. Autefage et al. (2015) reported a microarray study of hMSC after the treatment with growth medium conditioned and strontium doped bioactive glass. The results showed an upregulation of BMP-2 expression *in vitro* and *in vivo*, hence indicates that the promising commitment of hMSC toward osteoblastic lineage in the presence of SrBG as a treatment. In addition, the genome analysis also confirmed that the extract medium was able to upregulate TLR4, which is expressed in most human tissues, and activated the PI3K/Akt signaling pathway [308].

## 6 Prospects and challenges of bioactive coating systems for absorbable magnesium bone implants

The major issue of Mg implants is their high corrosion/degradation rate in the physiologic environment. Several strategies have been proposed to improve the Mg implant's corrosion resistance, including alloying and surface coatings. A functional coating that can both decrease the corrosion rate of Mg and increase bone regeneration would be a promising approach for the clinical translation of Mg implants. Hence, the coating should be biocompatible, corrosion-resistant, and bioactive. In this case, a composite coating of Sr substituted HA with collagen type I which can release recombinant human bone morphogenetic proteins 2, simvastatin, zoledronate can be example of an ideal candidate. It is also important to study the coating adhesion. The micro-arc oxidation (MAO) process can provide the strong bonding of the bioactive ceramic coating (e,g, CaP) to the Mg substrate, which produces dense and porous structures [[309], [310], [311], [312]]. This strong bonding is also beneficial for the attachment of a polymeric matrix loaded with growth factors, simvastatin, and zoledronate. Apart from *in vitro* studies, controlling the release amount and the efficiency of bone growth factors, simvastatin and zoledronate on bone regeneration *in vivo* are required to better understand the coating's biofunctionality. Three main factors play an important role in bone remodeling: cell signaling, oxygen tension, and stimulation [313]. The coatings in this review discussed only those that facilitate cell signaling for cell differentiation. Regulation of soluble growth factors by adjusting oxygen tension [314,315] and further study or combination of it with biophysical or biomechanical stimuli can be beneficial to accelerate bone regeneration [316].

## 7 Conclusion

The advancing knowledge in bone fracture and healing process combined with the advancing coating technology should enable us to design a well-suited bioactive magnesium implant that promotes the healing process within the optimum mechanical stability of the implant. The bone fracture healing process takes about 3–6 months through four consecutive stages, from inflammation to soft callus formation, hard callus formation, and finally, bone remodeling. Magnesium alloy bone implants have been proven to possess osteopromotive properties with known underlying mechanisms. Besides improving these properties, the bioactive coating can be exploited to accelerate the healing period to match the fast degradation of magnesium alloys. For this purpose, six bioactive agents have shown their high potential: hydroxyapatite, collagen type I, recombinant human bone morphogenetic proteins 2, simvastatin, zoledronate, and strontium. These agents can be used in combination to create optimum bioactivity. In addition to coating, alloying magnesium with calcium, zinc, copper, strontium, and manganese can potentially enhance the osteopromotive properties of magnesium substrate. Constant efforts and cooperation among materials scientists and clinicians are required to develop novel high-performance absorbable bone implants that speed up bone fracture repair.

## CRediT author statement

MNS: Investigation, Writing - original draft, Writing - review & editing, Visualization, Funding acquisition, Formal analysis. NIK: Investigation, Writing - review & editing. PS: Writing-review & editing. MR: Writing-review & editing QUA: Writing-review & editing. CS: Writing - review & editing. HH: Conceptualization, Writing - review & editing, Visualization, Supervision, Funding acquisition, Formal analysis.

## Declaration of competing interest

The authors declare that they have no known competing financial interests or personal relationships that could have appeared to influence the work reported in this paper.
